# Clinical Utility of Anti‐reflux Mucosal Intervention for Refractory Gastroesophageal Reflux Disease in Patients With a Hiatal Hernia

**DOI:** 10.1002/deo2.70424

**Published:** 2026-08-31

**Authors:** Masachika Saino, Haruhiro Inoue, Kei Ushikubo, Miyuki Iwasaki, Kazuki Yamamoto, Yohei Nishikawa, Ippei Tanaka, Hidenori Tanaka, Mayo Tanabe, Satoshi Abiko, Boldbaatar Gantuya, Manabu Onimaru, Shiro Oka

**Affiliations:** ^1^ Digestive Disease Center Showa Medical University Koto Toyosu Hospital Tokyo Japan; ^2^ Department of Gastroenterology Graduate School of Biomedical and Health Sciences Hiroshima University Hiroshima Japan

**Keywords:** endoscopy, gastroesophageal reflux, hernia, hiatal, minimally invasive surgical procedures, treatment outcome

## Abstract

**Background:**

Surgical fundoplication is the standard treatment for refractory gastroesophageal reflux disease (GERD) with large (≥3 cm) hiatal hernias; however, its invasiveness is problematic in older adults and high‐risk patients. Anti‐reflux mucosal intervention (ARMI) is a less invasive endoscopic alternative; however, patients with large hernias have previously been excluded from studies, limiting available evidence. We assessed the feasibility, short‐term clinical outcomes, and safety of ARMI in this population.

**Methods:**

We retrospectively analyzed consecutive patients with proton pump inhibitor (PPI)/potassium‐competitive acid blocker (P‐CAB)‐refractory GERD and sliding hiatal hernias ≥3 cm who underwent ARMI (anti‐reflux mucosectomy; anti‐reflux mucoplasty) between April 2024 and April 2025. GERD‐Health‐Related Quality of Life (GERD‐HRQL), GERD Questionnaire (GerdQ), and Frequency Scale for the Symptoms of GERD (FSSG) scores were compared before ARMI and 3 months afterward. PPI/P‐CAB discontinuation and treatment‐related adverse events were also assessed.

**Results:**

Ten patients (mean age 74.3 years; 80% women; hernia size 3–6 cm) were included, all with American Society of Anesthesiologists physical status ≥ II. At 3 months, mean GERD‐HRQL scores decreased from 21.1 to 4.6 (*p* = 0.002), FSSG from 22.6 to 6.2 (*p* = 0.004), and GerdQ from 10.9 to 4.6 (*p* = 0.008). PPI/P‐CAB was discontinued in seven patients. No clinically significant treatment‐related adverse events, including perforation, delayed bleeding, or dysphagia, occurred.

**Conclusions:**

ARMI was technically feasible, showed favorable short‐term outcomes in patients with refractory GERD and 3–6‐cm hiatal hernias, and may represent a less invasive option for older or high‐risk patients.

**Trial Registration:**

N/A.

**Abbreviations**
AET: acid exposure time
ARMI: anti‐reflux mucosal intervention
ARM‐P: anti‐reflux mucoplasty
ARMS: anti‐reflux mucosectomy
ASA‐PS: American Society of Anesthesiologists physical status
CO: cardiac opening
EPSIS: endoscopic pressure study integrated system
FSSG: Frequency Scale for the Symptoms of GERD
GERD‐HRQL: Gastroesophageal Reflux Disease Health‐Related Quality of Life
GerdQ: GERD Questionnaire
IGP: intragastric pressure
MII‐pH: multichannel intraluminal impedance–pH
mLA: modified Los Angeles
MNBI: mean nocturnal baseline impedance
P‐CAB: potassium‐competitive acid blocker
PPI: proton pump inhibitor
SH: sliding hernia.

## 1 Introduction

Surgical fundoplication procedures, such as Nissen and Toupet fundoplication, are the primary treatments for patients with gastroesophageal reflux disease (GERD) refractory to medical therapy [1, 2, 3]. Recently, anti‐reflux mucosal intervention (ARMI) has attracted attention as a relatively less invasive endoscopic alternative owing to its efficacy and safety [4, 5, 6].

The presence of a hiatal hernia promotes gastric content reflux into the esophagus and contributes to refractory GERD pathophysiology [7, 8]. ARMI does not directly repair the hiatal defect [9]; therefore, patients with large (≥3 cm) sliding hiatal hernias are considered unsuitable for endoscopic anti‐reflux therapy and excluded from many clinical studies [5, 10, 11]. Conversely, surgical fundoplication directly repairs the hiatal defect and is the standard treatment for patients with GERD and obvious hiatal herniation. However, surgical treatment is more invasive than endoscopic therapy and may not be suitable for high‐risk or older patients.

Recently, at our institution, we have encountered an increasing number of cases in which ARMI is selected as the initial treatment option for patients with refractory GERD and hiatal hernias ≥ 3 cm because surgery is considered difficult owing to advanced age or comorbidities. Therefore, we aimed to assess the feasibility, short‐term clinical outcomes, and safety of ARMI in patients with refractory GERD and hiatal hernias ≥ 3 cm, and to explore its potential role in this challenging patient population.

## 2 Methods

### 2.1 Study Design

This single‐center retrospective study was conducted at Showa Medical University Koto Toyosu Hospital using retrospectively collected electronic medical record data and patient‐completed questionnaires.

### 2.2 Outcome Measures

The primary outcome was change in symptom severity and health‐related quality of life from baseline to 3 months, assessed using the GERD‐Health‐Related Quality of Life questionnaire (GERD‐HRQL) [12], Frequency Scale for the Symptoms of GERD (FSSG) [13], and GERD Questionnaire (GerdQ) [14].

Secondary outcomes were proton pump inhibitor (PPI) and/or potassium‐competitive acid blocker (P‐CAB) therapy discontinuation or dose reduction at 3 months, adverse events, and changes in Endoscopic Pressure Study Integrated System (EPSIS) parameters.

### 2.3 Inclusion and Exclusion Criteria

Eligible patients were those with refractory GERD and a sliding hiatal hernia ≥ 3 cm who underwent ARMI between April 2024 and April 2025. Refractory GERD was defined as persistent symptoms despite ≥ 6 months of PPI or P‐CAB therapy. Patients with prior gastric surgery or previous ARMI were excluded.

### 2.4 Pre‐procedural Evaluation and Diagnosis of GERD

Following PPI/P‐CAB therapy discontinuation for at least 2 weeks, the GERD diagnosis and ARMI indication were determined according to the Lyon Consensus 2.0 [15] based on a detailed clinical history, upper gastrointestinal endoscopy, and 24‐h multichannel intraluminal impedance–pH (MII‐pH) monitoring, when available. EPSIS [16] was performed during upper gastrointestinal endoscopy as an adjunctive measure. Barium esophagography and high‐resolution esophageal manometry were performed to exclude primary esophageal motility disorders. Patients with functional heartburn were excluded, whereas those with reflux hypersensitivity were included. Functional heartburn was defined as acid exposure time (AET) < 4.0%, total reflux episodes <40/day, mean nocturnal baseline impedance (MNBI) >2500 Ω, and negative symptom–reflux association (symptom index <50% and symptom association probability <95%).

### 2.5 Interventions

**FIGURE 1:**
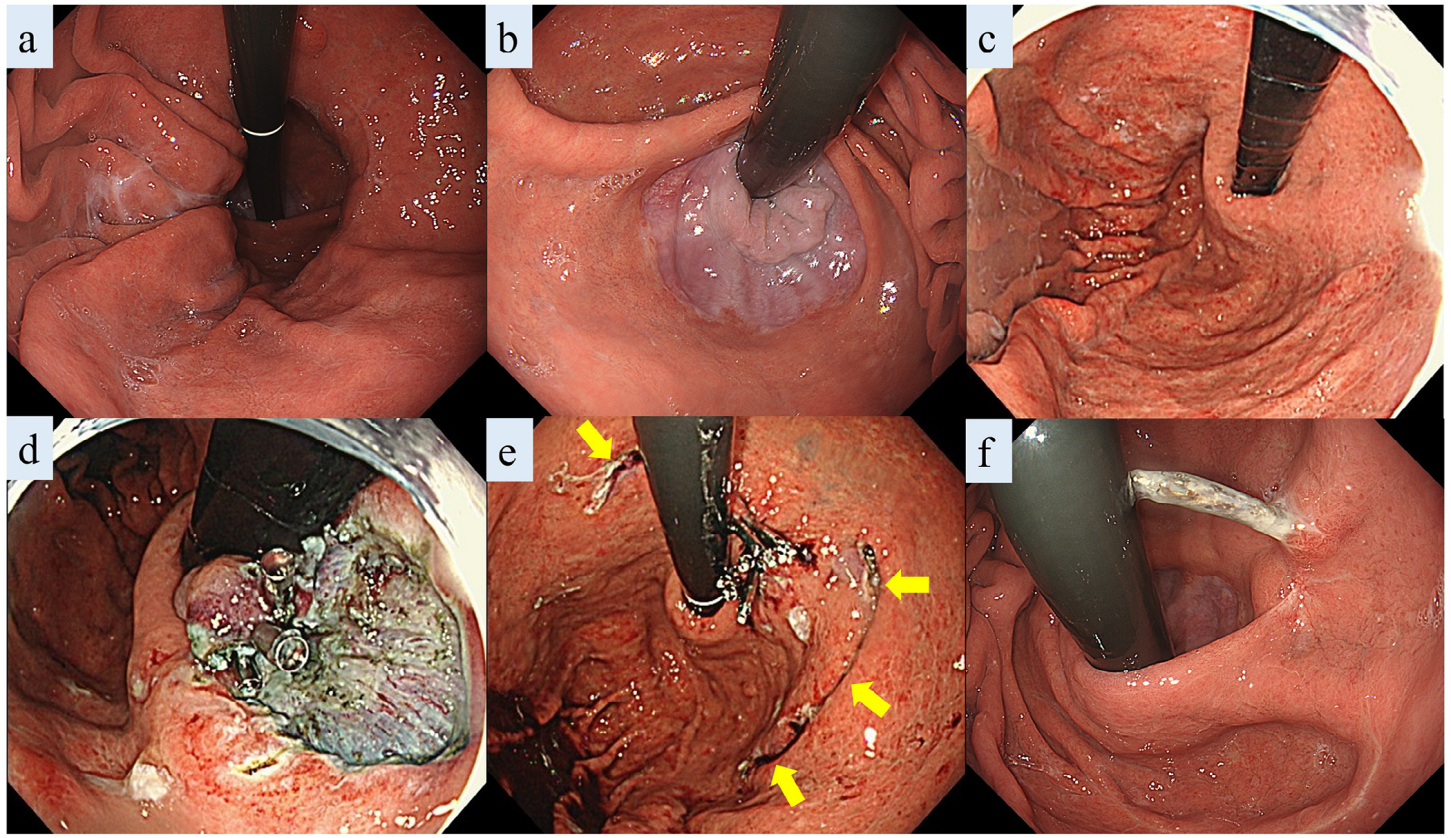
Representative endoscopic images before, during, and after anti‐reflux mucoplasty in the same patient with a 5‐cm sliding hiatal hernia. (a) Baseline retroflex view during preoperative endoscopy without general anesthesia or positive‐pressure ventilation, showing marked hiatal herniation. (b) Baseline retroflex view around the SCJ before treatment. (c) Immediately before ARM‐P under general anesthesia with positive‐pressure ventilation, showing reduction of the sliding hernia. (d) Endoscopic view after completion of submucosal dissection and valve creation during ARM‐P. (e) Final appearance after creation of counter mucosal incisions and mucosal defect closure. Yellow arrows indicate counter mucosal incisions. (f) Three‐month follow‐up retroflex view around the SCJ showing improvement in flap‐valve morphology compared with baseline. ARM‐P, anti‐reflux mucoplasty; SCJ, squamocolumnar junction.

All procedures were performed under general anesthesia with endotracheal intubation by an experienced endoscopist (Haruhiro Inoue). Anti‐reflux mucoplasty (ARM‐P) was generally selected when stable closure of the mucosal defect was considered technically feasible based on the esophagogastric junction configuration and endoscopic maneuverability; otherwise, anti‐reflux mucosectomy (ARMS) was selected. Technical details of ARMS and ARM‐P (including cases with valve creation, previously described as ARM‐P/V) followed previously published methods [17, 18, 19]. In ARM‐P, the mucosal defect was closed by a clip‐based technique incorporating MANTIS clips (Boston Scientific, Marlborough, MA, USA) or by endoscopic hand suturing using SutuArt (Olympus Medical Systems Corp., Tokyo, Japan). Figure 1 shows key endoscopic steps of ARM‐P in a representative case.

When complete mucosal resection was technically difficult, additional ablation was applied at the operator's discretion. In ARM‐P, counter mucosal incisions were added before closure to optimize tension control and prevent dehiscence [20] (Figure 1e).

Procedure time was defined as the interval from endoscope insertion to completion of mucosal resection for ARMS, mucosal defect closure for ARM‐P, or coagulation when ablation was performed. Antithrombotic agents were managed according to the Japan Gastroenterological Endoscopy Society guidelines for high‐risk endoscopic procedures [21, 22].

### 2.6 Periprocedural Management and Follow‐up

On postoperative day 1, all patients underwent upper gastrointestinal endoscopy to assess the treatment site and detect early complications. Symptoms, vital signs, and laboratory data were monitored during hospitalization. In the absence of complications, diet was advanced stepwise; patients were discharged after follow‐up endoscopy on postoperative days 4–5. P‐CAB 20 mg was continued for 30 days after treatment and then routinely discontinued in all patients. At 3 months, endoscopy and symptom score reassessment were performed off acid‐suppressive therapy; treatment was resumed only at the patient's request after reassessment.

### 2.7 Complication Definitions

Major intraprocedural bleeding was defined as bleeding requiring hemostatic intervention for >5 min or blood transfusion during the procedure. Delayed bleeding was defined as hematemesis, active bleeding on follow‐up endoscopy requiring endoscopic hemostasis, or a hemoglobin decrease of ≥2 g/dL from the pre‐treatment level. Perforation was defined as a defect confirmed endoscopically or on computed tomography during or after the procedure requiring endoscopic or surgical intervention. Stricture was assessed based on patient‐reported symptoms and follow‐up endoscopic findings. Clinically relevant stricture was defined as dysphagia requiring endoscopic dilation or dietary modification.

Other adverse events were classified according to the Clavien–Dindo classification [23]. Events of grade II or higher were defined as clinically significant adverse events.

### 2.8 Data Collection

Patient characteristics, endoscopic findings, adjunctive test results, treatment details, and post‐treatment outcomes were extracted from electronic medical records.

**FIGURE 2:**
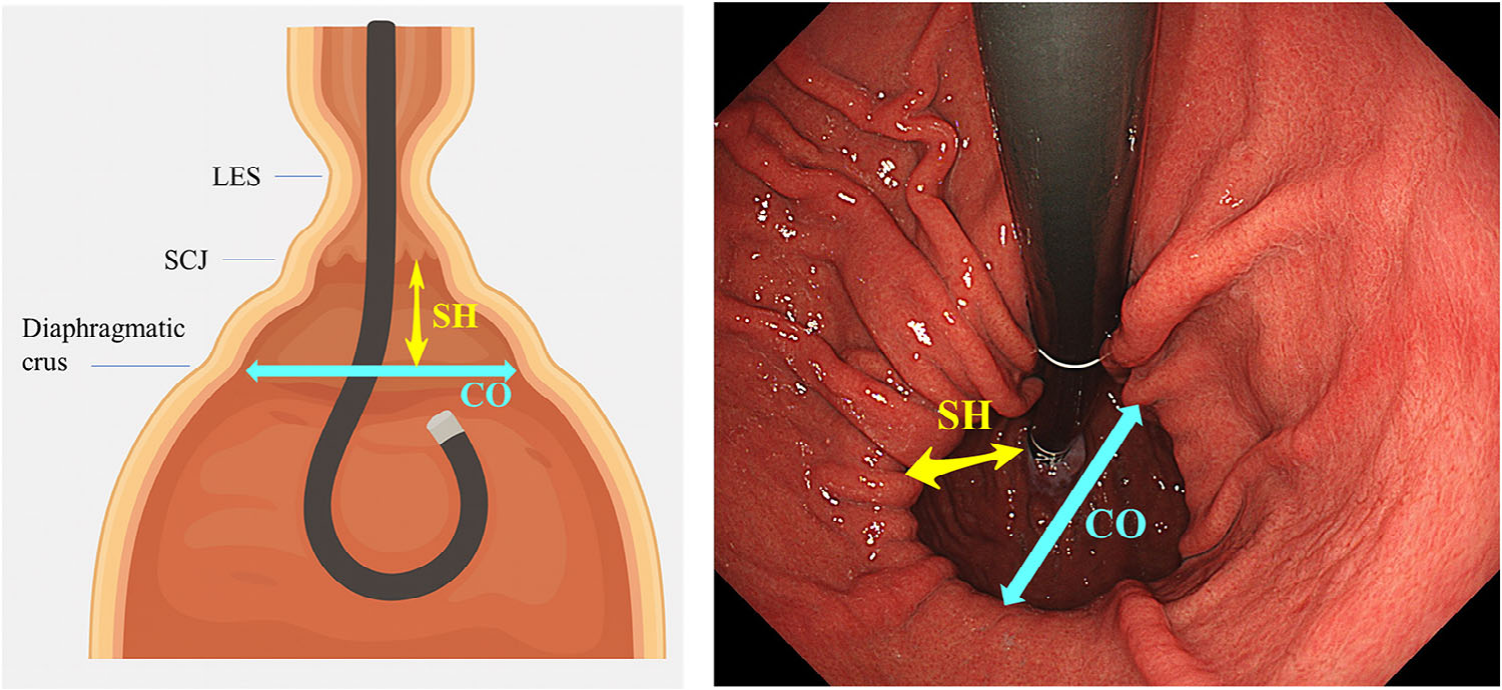
Schematic and endoscopic views of a sliding hiatal hernia showing measurement of the cardiac opening (CO) and sliding hernia (SH). LES, lower esophageal sphincter; SCJ, squamocolumnar junction.

Pre‐ and post‐treatment endoscopic images were independently evaluated by two endoscopists (Masachika Saino and Hidenori Tanaka) to assess hiatal hernia morphology using the cardiac opening–sliding hernia (CO–SH) classification [24] and gastroesophageal flap valve morphology using the Hill classification [25]. In the CO–SH classification, CO represents the diameter of the esophagogastric junction, whereas SH represents the distance from the diaphragmatic crus to the squamocolumnar junction, both measured in centimeters (Figure 2). CO and SH were recorded to the nearest centimeter. Interobserver agreement was assessed using the initial independent evaluations; discrepancies were subsequently resolved by consensus to determine the final assessments. Reflux esophagitis severity was graded using the modified Los Angeles (mLA) classification [26]. EPSIS parameters, including maximum intragastric pressure (IGP), pressure difference, and pressure gradient, were evaluated before and after treatment [27, 28]. For 24‐h MII‐pH monitoring, AET, total reflux episodes, and MNBI were collected. MNBI was measured at 5 cm above the proximal border of the lower esophageal sphincter.

Symptoms were assessed before and after treatment using self‐administered GERD‐HRQL, FSSG, and GerdQ questionnaires. Pre‐treatment scores were obtained while patients received PPI or P‐CAB therapy; post‐treatment scores were obtained at the first outpatient visit approximately 3 months after discharge while patients were off acid‐suppressive therapy.

### 2.9 Statistical Analysis

Continuous variables are presented as mean ± standard deviation or median (range), as appropriate. Pre‐ and post‐treatment endoscopic findings are summarized descriptively. Pre‐ and post‐treatment symptom scores and EPSIS parameters were compared using the Wilcoxon signed‐rank test, and mean changes with 95% confidence intervals were calculated descriptively. Paired analyses were performed using only patients with complete pre‐ and post‐treatment data for each variable; no imputation was performed. Statistical analyses were performed using JMP Pro 18 (SAS Institute Inc., Cary, NC, USA). All tests were two‐sided, with *p* < 0.05 considered significant. Interobserver agreement was assessed using the intraclass correlation coefficient [ICC(2,1)] for CO and SH and Cohen's *κ* for the Hill grade.

## 3 Results

### 3.1 Patients

Between April 2024 and April 2025, 10 consecutive patients with PPI/P‐CAB‐refractory GERD and a sliding hiatal hernia ≥ 3 cm underwent ARMI. All 10 patients met the eligibility criteria and were included in the final analysis.

### 3.2 Baseline Characteristics

Baseline characteristics are summarized in Table 1. The mean age was 74.3 years, 80% were women, and all patients had American Society of Anesthesiologists physical status (ASA‐PS) ≥ II. On baseline endoscopy after PPI/P‐CAB withdrawal, mLA grade was N/M in five patients, B in three, and D in two; median CO and SH were 4.5 and 3.0 cm, respectively. Baseline MII‐pH monitoring was performed in six patients; the remaining four did not provide consent. GERD phenotypes were classified as conclusive GERD in six patients, inconclusive GERD in one, reflux hypersensitivity in one, and unclassified in two patients without erosive esophagitis or MII‐pH data. In the two unclassified patients, a flat EPSIS pattern supported the indication for ARMI. Among patients with available MII‐pH data, the mean values for AET, total reflux episodes, and MNBI were 7.3%, 50, and 580 ohms, respectively. Post‐treatment MII‐pH monitoring was not performed because patients declined repeat testing after symptomatic improvement.

**TABLE 1:** Baseline patient characteristics and pre‐procedural assessment (*n* = 10).

Age, years	
Mean ± SD	74.3 ± 7.2
Range	57–85
Sex, *n* (%)	
Male	2 (20)
Female	8 (80)
BMI, mean ± SD, kg/m^2^	22.5 ± 2.9
Baseline acid‐suppressive therapy, *n* (%)	
Vonoprazan	9 (90)
Esomeprazole	1 (10)
ASA‐PS score, *n* (%)	
Class II	7 (70)
Class III	3 (30)
History of abdominal surgery, *n* (%)	5 (50)
Antithrombotic therapy, *n* (%)	1 (10)
Predominant symptom, *n* (%)	
Heartburn	4 (40)
Acid regurgitation	4 (40)
Globus	1 (10)
Chest pain	1 (10)
Endoscopic findings	
Esophagitis (mLA grade), *n* (%)	
Grade N or M	5 (50)
Grade B	3 (30)
Grade D	2 (20)
CO, median (range), cm	4.5 (3–7)
SH, median (range), cm	3 (3–6)
Hill classification, *n* (%)	
Grade III	3 (30)
Grade IV	7 (70)
24‐h impedance–pH monitoring (*n* = 6)	
Acid exposure time, mean (SD), %	7.3 (8.9)
Total number of reflux events, mean (SD)	50 (23)
MNBI, mean (SD), ohms	580 (354)
GERD phenotype, *n* (%)	
Conclusive GERD	6 (60)
Inconclusive GERD	1 (10)
Reflux hypersensitivity	1 (10)
Unclassified	2 (20)

### 3.3 Procedural Characteristics and Adverse Events

Procedural characteristics and adverse events are summarized in Table 2. The ARMI techniques included ARM‐P in eight patients, including five patients who underwent valve creation, and ARMS in two patients. The median procedure time was 46 min (range 27–120). No major intraprocedural bleeding, delayed bleeding, or perforation occurred. No patient developed dysphagia, required dietary modification, or showed endoscopic evidence of stricture during follow‐up. One patient experienced abdominal pain classified as Clavien–Dindo grade I; no clinically significant adverse events (Clavien–Dindo grade ≥ II) were observed. Perioperative management was individualized according to each patient's comorbidities and anesthetic risk; however, no patient required special precautions beyond standard care.

**TABLE 2:** Procedural characteristics and adverse events of anti‐reflux mucosal intervention (*n* = 10).

Treatment method, *n* (%)	
ARM‐P	8 (80)
ARMS	2 (20)
Anesthesia, *n* (%)	
General	10 (100)
Procedure time, min, median (range)	46 (27–120)
Adverse events, *n* (%)	
Major intraprocedural bleeding	0 (0)
Delayed bleeding	0 (0)
Perforation	0 (0)
Dysphagia	0 (0)
Others (Clavien–Dindo classification ≥ II)	0 (0)

### 3.4 Outcomes

**FIGURE 3:**
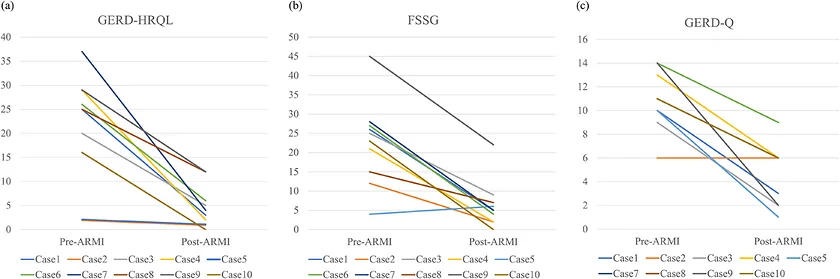
Individual trajectories of symptom scores at baseline and 3 months after anti‐reflux mucosal intervention (ARMI). (a) GERD‐HRQL, (b) FSSG, and (c) GerdQ (available in nine patients). GERD‐HRQL, Gastroesophageal Reflux Disease–Health‐Related Quality of Life; FSSG, Frequency Scale for the Symptoms of GERD; GerdQ, Gastroesophageal Reflux Disease Questionnaire.

Outcomes are summarized in Table 3. All five patients with baseline erosive esophagitis improved to mLA grade N. Interobserver agreement was good: ICC(2,1) was 0.878 for CO and 0.898 for SH, and Cohen's κ for Hill grade was 0.725. All interobserver differences were within 1 cm for CO and SH and within one grade for Hill grade. After consensus assessment, median CO and SH decreased from 4.5 to 3 cm and from 3 to 2 cm, respectively; Hill grade improved in four patients. Acid‐suppressive therapy was discontinued in seven patients, reduced by 50% in one, and continued in two. GERD‐HRQL, FSSG, and GerdQ scores decreased significantly (Table 3). Maximum IGP and pressure difference increased significantly, whereas pressure gradient did not increase significantly. GerdQ and EPSIS analyses included nine patients because of missing data. Individual trajectories are shown in Figure 3; GERD‐HRQL decreased in all patients, FSSG in nine, and GerdQ in eight.

**TABLE 3:** Outcomes after anti‐reflux mucosal intervention (*n* = 10).

Endoscopic findings	Pre‐ARMI	Post‐ARMI		
mLA grade, *n* (%)				
Grade N or M	5 (50)	10 (100)		
Grade B	3 (30)	0 (0)		
Grade D	2 (20)	0 (0)		
CO, median (range), cm	4.5 (3–7)	3 (1–5)		
SH, median (range), cm	3 (3–6)	2 (0–5)		
Hill classification, n (%)				
Grade II	0 (0)	2 (20)		
Grade III	3 (30)	3 (30)		
Grade IV	7 (70)	5 (50)		
PPI/P‐CAB after ARMI, *n* (%)				
Discontinuation		7 (70)		
50% dose reduction		1 (10)		
Continuation		2 (20)		
Symptom scores	Pre‐ARMI	Post‐ARMI	Change (95% CI)	*p*‐value
GERD‐HRQL, mean (SD)	21.1 (11.5)	4.6 (4.3)	−16.5 (−23.7 to −9.3)	**0.002**
FSSG, mean (SD)	22.6 (11.0)	6.2 (6.1)	−16.4 (−22.5 to −10.3)	**0.004**
GerdQ (*n* = 9), mean (SD)	10.9 (2.6)	4.6 (2.7)	−6.3 (−8.9 to −3.8)	**0.008**
EPSIS parameters (*n* = 9)	Pre‐ARMI	Post‐ARMI	Change (95% CI)	*p*‐value
Maximum IGP, mean (SD), mmHg	12.3 (4.9)	16.8 (5.4)	4.5 (0.4 to 8.5)	**0.031**
Pressure difference, mean (SD), mmHg	6.2 (5.3)	9.3 (5.1)	3.1 (0.5 to 5.7)	**0.023**
Pressure gradient, mean (SD), mmHg/s	0.15 (0.14)	0.21 (0.15)	0.06 (−0.03 to 0.15)	0.164

Change was calculated as post‐ARMI minus pre‐ARMI.

### 3.5 Individual Case Assessment

Individual case details are summarized in Table S1. Despite variability in baseline mLA grade and hiatal hernia size, symptom improvement was observed in the two patients with baseline mLA grade D and in those with relatively large sliding hiatal hernias (≥5 cm). In Case 5, FSSG increased slightly at 3 months (4–6), and P‐CAB therapy was continued; however, GERD‐HRQL and GerdQ decreased. In Case 2, GerdQ was unchanged, whereas GERD‐HRQL and FSSG decreased, allowing the discontinuation of acid‐suppressive therapy.

## 4 Discussion

ARMI was technically feasible without major complications in patients with refractory GERD and 3–6‐cm sliding hiatal hernias. At 3 months, all three symptom scores improved significantly, and acid‐suppressive therapy was discontinued or reduced in eight patients.

In patients with 3–6‐cm sliding hiatal hernias, ARMI median procedure time was 46 min. A comparative study reported mean procedure times of 36 min for ARMS and 101 min for laparoscopic fundoplication, indicating a relatively short procedure time for endoscopic therapy [29]. Our median procedure time was within the range reported in a systematic review of ARMS and was shorter than those reported in pilot studies of ARM‐P and ARM‐P/V [5, 18, 19]. Relatively large sliding hiatal hernias may increase the technical difficulty of endoscopic therapy because mediastinal displacement of the esophagogastric junction compromises visualization and restricts the working space. Positive‐pressure ventilation under general anesthesia partially reduced the SH during the procedure and may have supported favorable visualization and maneuverability (Figure 1a,c). Other practical considerations include performing the mucosal intervention immediately distal to the esophagogastric junction rather than at the hiatal orifice and, when ARM‐P is selected, reducing closure tension through counter mucosal incisions and avoiding forceful closure. Procedure time was not markedly prolonged compared with that reported in previous studies, suggesting that ARMI was technically feasible even in selected patients with hiatal hernia. From a practical perspective, general anesthesia with endotracheal intubation may be preferable for ARMI in patients with sliding hiatal hernias larger than 3 cm; however, the feasibility and safety of ARMI under conscious sedation remain to be established.

In the present small series, no clinically significant procedure‐related adverse events, including delayed bleeding and dysphagia, were observed after ARMI. GERD is a benign condition; hence, safety and invasiveness are key considerations when selecting a treatment strategy. A recent systematic review revealed that postoperative dysphagia occurred in 22.4% of patients at 1 year and 45.3% at 10 years after surgical fundoplication; reoperation was required in 6.7% at 1 year and 16.3% at 10 years, mainly owing to persistent dysphagia and recurrent reflux symptoms [30]. Conversely, a systematic review of ARMS reported dysphagia/stricture in 11.4% and delayed bleeding in 5.0%; these events were generally manageable with endoscopic treatment [5]. Moreover, relatively new techniques such as ARM‐P, which include the closure of the ulcer base to reduce the effective mucosal defect, are expected to further mitigate these risks. We did not compare ARMI directly with surgical fundoplication. However, these prior data and our findings suggest that ARMI may represent a relatively less invasive endoscopic option in selected patients.

The study population comprised older and clinically high‐risk individuals, with all patients classified as ASA‐PS ≥ II. A large analysis using a U.S. surgical registry revealed that the 30‐day perioperative complication rate after laparoscopic fundoplication increased significantly in older patients and identified age ≥ 70 years and ASA‐PS ≥ III as independent risk factors for complications [31]. Additionally, a direct comparison between ARMS and Nissen fundoplication reported a relatively short postoperative hospital stay and early return to daily activities in the ARMS group [29]. Among adults aged ≥ 68 years, relatively short hospitalization is associated with a reduced risk of decline in activities of daily living and mobility [32]. Therefore, treatment strategies that reduce hospitalization duration may offer an important advantage for older patients.

In our cohort of patients with refractory GERD and a 3–6‐cm sliding hiatal hernia, GERD‐HRQL, FSSG, and GerdQ scores improved significantly; PPI/P‐CAB therapy could be discontinued in seven patients. Nevertheless, a slight increase or no change in a single symptom score was observed in a few patients, possibly because their baseline symptom scores were relatively low, resulting in only a small absolute change. Traditionally, the indication for ARMI has been limited to patients with relatively short hernias (e.g., < 2–3 cm), whereas those with relatively advanced SHs have generally been considered candidates for surgical fundoplication [33]. However, a limited number of case reports have described the clinical benefit of ARMI even in patients with advanced hiatal hernias [34, 35, 36]. A plausible explanation for the favorable outcomes observed in the present study, despite the absence of hiatal defect repair, is functional reinforcement of the anti‐reflux barrier through recreating a flap‐valve around the esophagogastric junction proximal to the hernia orifice (Figure 1f). Additionally, EPSIS parameters showed significant increases in maximum IGP and pressure difference after treatment, suggesting a possible improvement in anti‐reflux function [28]. These findings suggest that ARMI offers a certain degree of clinical benefit even in patients with a sliding hiatal hernia ≥ 3 cm and support the need for further investigation.

This study has some limitations. First, it was a small, single‐center retrospective study; the cohort was skewed toward older women with comorbidities, which may have introduced selection bias. Second, all procedures were performed by an experienced operator at a high‐volume center; the cohort was predominantly treated with closure‐based techniques such as ARM‐P rather than with resection‐based approaches, limiting the generalizability of these findings to other settings and ARMI techniques. Third, no control group was available; the follow‐up period was limited to 3 months. Therefore, comparative effectiveness, long‐term durability, overall clinical success, and the potential role of ARMI as an alternative to surgical fundoplication in patients with large hiatal hernias could not be adequately determined. Future prospective comparative studies with extended follow‐up are needed to evaluate these issues. Fourth, in two patients without erosive esophagitis or MII‐pH data, GERD could not be objectively confirmed despite adjunctive information provided by a flat EPSIS pattern, and functional heartburn could not be completely excluded.

Despite these limitations, our findings suggest that ARMI is beneficial in selected patients with refractory GERD and advanced sliding hiatal hernias and provide a basis for future studies on patient selection and further clinical investigation.

## Author Contributions

**Masachika Saino**: conceptualization, data curation, formal analysis, investigation, methodology, project administration, visualization, and writing – original draft. **Haruhiro Inoue**: conceptualization, methodology, resources, supervision, and writing – review and editing. **Kei Ushikubo**: investigation, data curation, and writing – review and editing. **Miyuki Iwasaki**: investigation, data curation, and writing – review and editing. **Kazuki Yamamoto**: investigation, data curation, and writing – review and editing. **Yohei Nishikawa**: investigation, data curation, and writing – review and editing. **Ippei Tanaka**: investigation, data curation, and writing – review and editing. **Hidenori Tanaka**: investigation, data curation, validation, and writing – review and editing. **Mayo Tanabe**: investigation, data curation, and writing – review and editing. **Satoshi Abiko**: investigation, data curation, and writing – review and editing. **Boldbaatar Gantuya**: investigation, data curation, and writing – review and editing. **Manabu Onimaru**: investigation, data curation, and writing – review and editing. **Shiro Oka**: supervision and writing – review and editing. All authors approved the final version of the manuscript.

## Funding

This research received no external funding. The authors have nothing to report.

## Ethics Statement

**Approval of the research protocol**: This study was approved by the Institutional Review Board of Showa Medical University School of Medicine (No. 2025‐0015) and was conducted in accordance with the Declaration of Helsinki.

## Consent

Informed consent was obtained using an opt‐out approach.

## Conflicts of Interest

Haruhiro Inoue serves as an advisor for Olympus Corporation and Top Corporation. He has also received educational grants from Olympus Corporation and Takeda Pharmaceutical Co. The other authors declare no conflicts of interest.

## Supporting information




**FILE S1**: Supplementary_material_R3.docx
**TABLE S1**: Details of individual cases, including baseline characteristics, GERD phenotype, endoscopic findings, treatment method, symptom‐score changes, and post‐treatment acid‐suppressive therapy status.
