# Supplementary material for: Clinical Utility of Anti‐reflux Mucosal Intervention for Refractory Gastroesophageal Reflux Disease in Patients With a Hiatal Hernia
Source: DEN Open. 2026 Aug 31;7(1):e70424. doi: 10.1002/deo2.70424 (PMC13528452; doi:10.1002/deo2.70424)
Supplement: Supplementary file 1 [file DEO2-7-e70424-s001.docx]

**Supplementary Table 1. Details of individual cases**

| No. | Age | Sex | ASA-PS | Main comorbidities | mLA  Grade | GERD phenotype | CO  (cm) | SH  (cm) | Hill  classification | Treatment  (closure method) | GERD-HRQL | FSSG | GerdQ | PPI/P-CAB |
| --- | --- | --- | --- | --- | --- | --- | --- | --- | --- | --- | --- | --- | --- | --- |
| 1 | 70s | F | II | Hypertension;  chronic kidney disease | N | Conclusive GERD | 5→4 | 5→4 | IV→IV | ARM-P (clips) | 25→3 | 26→5 | 10→3 | Discontinuation |
| 2 | 70s | F | II | Prior lung cancer surgery; prior inguinal hernia repair | N | Unclassified | 7→5 | 6→5 | IV→IV | ARM-P (clips) | 2→1 | 12→2 | 6→6 | Discontinuation |
| 3 | 70s | F | II | Prior appendectomy; osteoporosis | B | Conclusive GERD | 5→4 | 3→3 | IV→IV | ARM-P (clips) | 20→5 | 25→9 | 9→2 | Discontinuation |
| 4 | 70s | F | II | Hypertension;  diabetes mellitus | B | Conclusive GERD | 5→3 | 3→2 | IV→III | ARM-P (clips) | 29→2 | 21→2 | 13→6 | 50% dose reduction |
| 5 | 70s | F | II | Hypertension; osteoporosis | B | Conclusive GERD | 3→2 | 3→1 | IV→III | ARM-P (clips) | 2→1 | 4→6 | 10→1 | P-CAB 20 mg |
| 6 | 50s | M | III | Spinocerebellar degeneration; vasospastic angina | N | Reflux hypersensitivity | 4→2 | 3→1 | III→II | ARM-P (suturing) | 26→6 | 27→4 | 14→9 | Discontinuation |
| 7 | 80s | M | III | Prior myocardial infarction; ITP | N | Unclassified | 4→2 | 3→1 | III→III | ARM-P (suturing) | 37→4 | 28→5 | 11→6 | Discontinuation |
| 8 | 70s | F | II | Hypertension; pancreatitis | M | Inconclusive GERD | 4→3 | 4→3 | IV→IV | ARM-P (clips) | 25→12 | 15→7 | NA | Discontinuation |
| 9 | 60s | F | III | SSc with pulmonary hypertension; COPD | D | Conclusive GERD | 3→1 | 3→0 | III→II | ARMS | 29→12 | 45→22 | 14→2 | P-CAB 20 mg |
| 10 | 80s | F | II | Prior surgery for strangulated ileus; cerebral aneurysm | D | Conclusive GERD | 5→3 | 3→2 | IV→IV | ARMS | 16→0 | 23→0 | 11→6 | Discontinuation |

Values for CO, SH, Hill classification, GERD-HRQL, FSSG, and GerdQ are shown as pre→post.

**Abbreviations:** F, female; M, male; ASA-PS, American Society of Anesthesiologists physical status; ITP, idiopathic thrombocytopenic purpura; SSc, systemic sclerosis; COPD, chronic obstructive pulmonary disease; mLA, modified Los Angeles classification (N = none, M = minimal change, B = grade B, D = grade D); CO, cardiac opening; cm, centimeters; SH, sliding hernia; ARMI, anti-reflux mucosal intervention; ARM-P, anti-reflux mucoplasty; ARMS, anti-reflux mucosectomy; GERD-HRQL, Gastroesophageal Reflux Disease Health-Related Quality of Life; FSSG, Frequency Scale for the Symptoms of GERD; GerdQ, GERD Questionnaire; NA, not available; PPI, proton pump inhibitor; P-CAB, potassium-competitive acid blocker.
